# Decreased expression of chromodomain helicase DNA-binding protein 9 is a novel independent prognostic biomarker for colorectal cancer

**DOI:** 10.1590/1414-431X20187588

**Published:** 2018-07-23

**Authors:** Li Xu, Hui Peng, Xiao-Xu Huang, Ya-Bin Xia, Kai-Feng Hu, Zheng-Ming Zhang

**Affiliations:** 1 Department of General Surgery, the First Affiliated Yijishan Hospital of Wannan Medical College, Wuhu, Anhui, China; 2 Administration Office of Hospital Admission and Discharge, the First Affiliated Yijishan Hospital of Wannan Medical College, Wuhu, Anhui, China

**Keywords:** Chromodomain helicase DNA-binding protein 9, Colorectal cancer, Immunohistochemical analysis, Biomarker, Prognosis

## Abstract

Previous studies suggested that chromodomain helicase DNA-binding proteins (CHDs), including CHD 1–8, were associated with several human diseases and cancers including lymphoma, liver cancer, colorectal cancer, stomach cancer, etc. To date, little research on CHD 9 in human cancers has been reported. In this study, we assessed the prognostic value of CHD 9 in patients with colorectal cancer (CRC). We screened for CHD 9 expression using immunohistochemical analysis in 87 surgical CRC specimens and found that the expression was upregulated in 81.5% of the cases, while 7.4% were decreased; in the remaining 11.1% of the cases, levels were not altered. Kaplan-Meier analysis showed that patients with high CHD 9 expression had better prognosis than those with low CHD 9 expression (54.5 *vs* 32.1%, P=0.034). Subsequently, Cox multi-factor survival regression analysis revealed that expression of CHD 9 protein was an independent predictor for CRC, with a hazard ratio of 0.503 (P=0.028). In addition, we found that CHD 9 expression was positively correlated with MSH2 (r_s_=0.232, P=0.036). We speculated that CHD9 might be a putative tumor suppressor gene, and could inhibit the development of CRC by participating in DNA repair processes. Our findings suggest that CHD 9 could be a novel prognostic biomarker and a therapeutic target for CRC. Further studies are needed to detect the effect of CHD 9 on cellular function and the expression of mismatch repair genes.

## Introduction

Colorectal cancer (CRC) is one of the most common gastrointestinal tumors. It has become the third leading cause of cancer-related death worldwide (1). In recent years, with the rapid development of the Chinese economy and change of diet, CRC has become one of the malignant tumors with the fastest rising incidence in China (2,3). Although it has become routine to screen for the disease and new technologies are being developed, the prevention, treatment, and prognosis of CRC remain a significant problem in the global public health field. Therefore, a better understanding of the molecular mechanisms involved in the progression of CRC is crucial to explore novel therapeutic targets for CRC treatment.

The chromodomain helicase DNA-binding proteins (CHDs) are a family of nine members named CHD 1–9, which act as regulators of chromatin remodeling process and gene expression in humans. Chromatin remodeling is the dynamic modification of chromatin architecture to allow access of condensed genomic DNA to the regulatory transcription machinery proteins, playing a critical role in regulating gene expression during the developmental period. All CHD proteins contain two basal tandem chromo domains and different additional domains. Thus, the CHD family could be divided into three sub-families according to the additional domains and features: CHD 1–2, CHD 3–5, and CHD 6–9 (4,5). There is emerging evidence suggesting that CHDs might contribute to a broad spectrum of human diseases and cancers, including lymphoma, liver cancer, colorectal cancer, stomach cancer, etc (6–9). The third sub-family of CHD enzymes are orthologs of the Drosophila Kismet enzyme and are characterized by the Brahma and Kismet domains at C termini. The mutant of CHD 7 and 8 could lead to the distinct disease states of CHARGE syndrome (10) and autism spectrum disorders (11). However, to date, few studies on the CHD 9 protein in human disease have been reported.

Previous studies have shown that CHD 9 has a certain mutation rate in high-level microsatellite instability (MSI-H) CRC, but neither its role in CRC nor its effect on prognosis has yet been reported (5). Approximately 12–15% CRC have deficient DNA mismatch repair, which is characterized in the tumor by MSI (12). Therefore, it is of great importance to study the specific mechanism of CHD 9 in CRC and its effect on the prognosis of CRC. Here, we evaluated CHD 9 expression in CRC in Chinese patients. To our knowledge, this is the first study to assess the prognostic value of CHD 9 in CRC.

## Material and Methods

A total of 87 patients with CRC (44 females, 42 males, 1 lost information) who had undergone surgical procedures at Yijishan Hospital of Wannan Medical College between July 2006 and May 2007 were enrolled in the study. The patients' tissue microarray contained well-documented clinical-pathological information, including patients' gender, age, tumor size, tumor differentiation, stage, N stage, distant metastasis, and clinical stage (Table 1). Patients ranged in age from 24 to 90 (means±SD, 69.51±11.01). Mean tumor size was 5.7 cm (range 1.5–15.0).

**Table 1.** Correlation between clinical data and CHD 9 expression in colorectal cancer.

Clinical parameters	Sample size			CHD 9 carcinoma score (means±SD)	Test statistic (t/F)	P
	N	Total	Loss			
Gender		85	2			
Male	43			8.37±1.92	–0.27	0.792
Female	42			8.48±1.70		
Age		81	6			
>60 years	66			8.48±1.79	–0.42	0.677
≤60 years	15			8.27±1.98		
Tumor size		85	2			
>5cm	40			8.30±1.47	0.48	0.631
≤5cm	45			8.49±2.05		
Pathological grading		86	1			
I	3			6.67±2.31	2.19	0.118
II	43			8.70±1.68		
III	40			8.25±1.62		
Tumor		79	8			
T1-T2	8			8.25±1.98	–0.43	0.668
T3-T4	71			8.54±1.76		
Node		86	1			
N0	55			8.47±1.92	0.65	0.525
N1	22			8.55±1.26		
N2	9			7.78±2.11		
Metastasis		86	1			
M0	84			8.43±1.81	–0.33	0.740
M1	2			8.00±0.00		
Clinical staging		85	2			
Stage I	8			8.25±1.98	0.06	0.980
Stage II	46			8.43±1.88		
Stage III	29			8.34±1.61		
Stage IV	2			8.00±0.00		

Student's *t*-test or one-way ANOVA.

The patients' CRC tissue microarray (HCol-Adel180sur-06) was made by Shanghai Outdo Biotech Co., Ltd. (China). The CRC microarray was constructed by formalin-fixed tissue samples embedded in paraffin from 87 patients. The typical pathological sites on HE slices were labeled by pathologists, then drilled on the blank recipient paraffin (diameter was 1.5 mm) using tissue microarray instrument. All of the 87 samples with their adjacent para-carcinoma tissues were collected 1.5 cm away from the cancer tissue.

The follow-up time of CRC patients was August 2015, ranging from 87 to 97 months. The result of statistical analysis showed that during the follow-up time, 56 of the 87 patients died of CRC, and the other 31 patients were still alive, with the median follow-up time of about 92 months. All patients were diagnosed as CRC and received no treatment before surgery.

This study was approved by the Ethics Committee of the Yijishan Hospital of Wannan Medical College, and informed consent was obtained from all the participants.

### Immunohistochemistry

Two-step immunohistochemistry assay was used in this study. Tissue sections were treated with EDTA buffer under high pressure at high temperature to retrieve antigen. Then, sections were incubated with primary antibody named anti-CHD9 (1:3000, 13402–1-AP, Proteintech, USA) at 4°C overnight. Sections were then washed with PBS after incubating with secondary antibody (HRP-labeled anti-rabbit antibody; DAKO, Denmark). Samples were visualized using diaminobenzidine system and hematoxylin re-dying, and analyzed under microscope (OLYMPUS CX41, Japan). Three random high-magnification fields of each specimen were chosen under optical microscope and more than 300 cells were selected for the evaluation. The CHD 9 expression was scored and grouped by positive staining rate and intensity. The positive staining rate was defined according to the proportion of stained cancer cells: “Negative” is 0, “1–25%” is 1, “26–50%” is 2, “51–75%” is 3, “76–100%” is 4. The score for staining intensity was defined as follows: “Negative” is 0, “1+” is 1, “2+” is 2, “3+” is 3. Thus, patients were divided into low expression (≤8) and high expression (>8) groups according to the scores after multiplying “positive staining rate score” by the “staining intensity score”.

### Statistical analysis

Student's *t*-test or one-way ANOVA was used to assess the association between CHD 9 expression and various clinic-pathological parameters and molecular markers. Spearman's correlation analysis was used to calculate the relationship between the CHD9 expression and the several mismatch repair genes including MLH1, MSH2, MSH6, and PMS2. The survival rate was calculated with the Kaplan-Meier method and differences were evaluated using the log-rank test. Finally, statistically significant variables in univariate analysis were included in COX multivariate regression survival analysis. In all tests, two-sided P values <0.05 were considered statistically significant.

## Results

**Figure 1.**
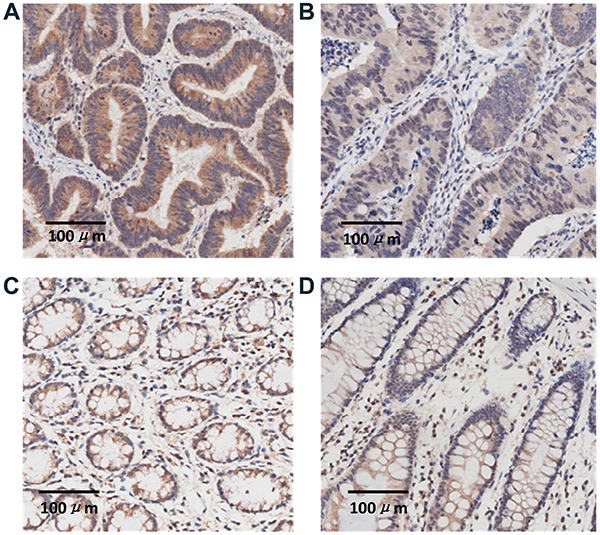
Representative immunohistochemistry images of CHD 9 expression in colorectal cancer tissues and para-carcinoma tissues: *A*, high CHD 9 expression in tumor; *B*, low CHD 9 expression in tumor; *C*, high CHD 9 expression in adjacent tissues; *D*, low CHD 9 expression in adjacent tissues (Magnification: ×200; bar: 100 μm).

Representative immunohistochemistry images are shown in Figure 1. The CHD 9 expression was upregulated in 81.5% of the cases, while 7.4% of the cases showed decreased expression. CHD 9 expression was not altered in the remaining 11.1%. Spearman's correlation analysis showed that the expression of CHD 9 was neither correlated with age, gender, tumor size, nor the clinical classification or pathological grading (all P>0.05). The results are shown in Table 1.

Spearman's correlation analysis was used to assess the relationships between CHD 9 expression and mismatch repair genes including MLH1, MSH2, MSH6, and PMS2. CHD9 expression was positively correlated with MSH2 (r_s_=0.232, P=0.036) (Table 2).

**Table 2.** Correlation analysis of CHD 9 expression and mismatch repair genes.

Variables	Correlation	CHD9 carcinoma score	MLH1 carcinoma	MSH2 carcinoma	MSH6 carcinoma	PMS2 carcinoma
CHD 9 carcinoma score	r_s_	1.000	–0.076	0.232	0.113	0.154
	P		0.491	0.036	0.301	0.163
	N	86	84	82	86	83
MLH1 carcinoma	r_s_	–0.076	1.000	0.569	0.468	0.437
	P	0.491		<0.001	<0.001	<0.001
	N	84	88	84	88	86
MSH2 carcinoma	r_s_	0.232	0.569	1.000	0.676	0.276
	P	0.036	<0.001		<0.001	0.012
	N	82	84	85	85	83
MSH6 carcinoma	r_s_	0.113	0.468	0.676	1.000	0.290
	P	0.301	<0.001	<0.001		0.006
	N	86	88	85	90	87
PMS2 carcinoma	r_s_	0.154	0.437	0.276	0.290	1.000
	P	0.163	<0.001	0.012	0.006	
	N	83	86	83	87	87

r_s_: spearman correlation coefficient; P: P value; N: number of subjects.

**Figure 2.**
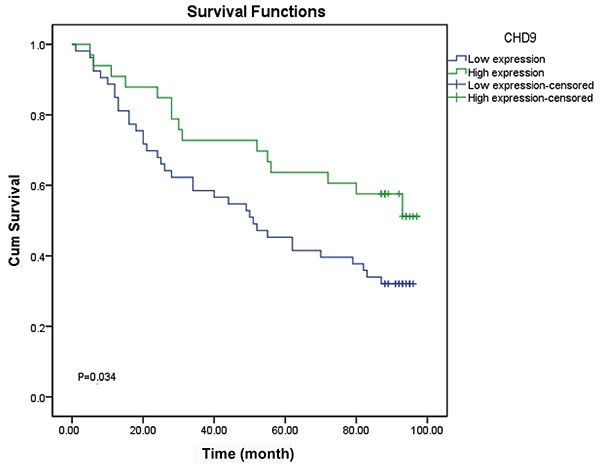
Correlation of CHD 9 expression and the prognosis of colorectal cancer.

Kaplan-Meier method and the log-rank test showed that CRC patients with high expression of CHD 9 had a significantly better prognosis than those with low level (54.5 *vs* 32.1%, P=0.034). The results are shown in Figure 2.

COX survival analysis showed that CHD 9 expression was an independent predictor for CRC, with a hazard ratio (HR) of 0.503 (P=0.028). The results are reported in Table 3.

**Table 3.** COX multivariate regression analysis of the independent predictors of CHD 9 in colorectal cancer patients.

Variables	B	SE	Wald	P value	HR
CHD 9 carcinoma score	–0.688	0.314	4.801	0.028	0.503
Node	0.463	0.361	1.642	0.200	1.589
Metastasis	1.063	0.859	1.532	0.216	2.896
Clinical staging	0.267	0.399	0.449	0.503	1.306

SE: standard error; HR: hazard ratio.

## Discussion

CHD protein family is extremely important in regulating gene expression and chromosome structure modification. CHD protein expression is associated with many diseases, such as lymphoma, liver cancer, colon cancer, gastric cancer, etc (4,6,13). CHD 9 has a certain mutation rate in the CRC of MSI-H, but its specific mechanism in CRC and the effects on prognosis have not yet been reported (5).

Based on previous research, MSI refers to repeated DNA nucleotide units in microsatellites, which arises in tumors when the function of mismatch repair is decreased by the inactivation of any one of the four mismatched repair genes: MLH1, MSH2, MSH6, and PMS2 (14 [Bibr B15] [Bibr B16] [Bibr B17] 14). About 12–15% CRC have deficient DNA mismatch repair and the MSI-H phenotype, although the majority of colorectal cancers develop via a chromosomal instability pathway and follow the classical adenoma-carcinoma sequence of tumor progression (10,15–18).

The present study demonstrated that CHD 9 expression was positively correlated with MSH2. Previous studies have shown that DNA damage repair mechanism is a critical pathway to ensure genome stability. CHDs are correlated with DNA damage repair: CHD 4 acts as a key regulator of homologous recombination repair through binding to BRIT1 (19). CHD 2, 3, 5, and 6 are also associated with DNA repair, the maintenance of genomic stability and/or cancer prevention (20,21). Thus, we assumed that CHD 9 might inhibit the development of colorectal cancer by participating in the DNA repair process. Our study assessed for the first time the relationship between CHD 9 remodeling protein and CRC progression. The results showed that patients with high CHD 9 expression had better prognosis and that CHD 9 expression was an independent predictor for colorectal cancer. Our findings indicated that the CHD 9 is a putative tumor suppressor gene and a new potential prognostic biomarker in CRC.

In conclusion, our research showed a correlation between CHD 9 expression and CRC prognosis, as well as the potential pathways of DNA mismatch repair process. Further study, such as examining the effect of CHD 9 expression on cellular function by knocking out or expressing CHD 9 genes in CRC cell lines, will be done to explore the tumor suppressor mechanism of CHD9.
